# Coded Distributed Computing Under Combination Networks

**DOI:** 10.3390/e27030311

**Published:** 2025-03-16

**Authors:** Yongcheng Yang, Yifei Huang, Xiaohuan Qin, Shenglian Lu

**Affiliations:** 1 Key Lab of Education Blockchain and Intelligent Technology, Ministry of Education, Guangxi Normal University, Guilin 541004, China; yongchengyang@stu.gxnu.edu.cn (Y.Y.); huangyifei59@163.com (Y.H.); lsl@gxnu.edu.cn (S.L.); 2 School of Science, Guilin University of Aerospace Technology, Guilin 541004, China; 3 State Key Laboratory for Chemistry and Molecular Engineering of Medicinal Resources, School of Chemistry and Pharmaceutical Science, Guangxi Normal University, Guilin 541004, China

**Keywords:** coded computing, communication, MapReduce, combination network

## Abstract

Coded distributed computing (CDC) is a powerful approach to reduce the communication overhead in distributed computing frameworks by utilizing coding techniques. In this paper, we focus on the CDC problem in (H,L)-combination networks, where *H* APs act as intermediate pivots and K=HL workers are connected to different subsets of *L* APs. Each worker processes a subset of the input file and computes intermediate values (IVs) locally, which are then exchanged via uplink and downlink transmissions through the AP station to ensure that all workers compute their assigned output functions. In this paper, we first novelly characterize the transmission scheme for the shuffle phase from the view point of the coefficient matrix and then obtain the scheme by using the Combined Placement Delivery Array (CPDA). Compared with the baseline scheme, our scheme significantly improves the uplink and downlink communication loads while maintaining the robustness and efficiency of the combined multi-AP network.

## 1. Introduction

The rapid growth of computationally intensive applications has driven considerable research into efficient distributed computing frameworks. MapReduce [1,2] and its evolution Apache Spark [3] have significant advantages in computational tasks dealing with massive datasets, where the amount of data can usually reach tens of terabytes. The MapReduce framework distributes computation tasks to multiple nodes, each storing a subset of the dataset. The computation process is decomposed into a set of “map” functions and “reduce” functions. Each map function processes a batch of data to generate intermediate values (IVs), which serve as inputs to the reduce functions. The overall process comprises three main phases: map, shuffle, and reduce phases. The nodes compute the map functions on their locally stored data, generate output IVs during the map phase, and exchange their computed IVs to ensure that each reduce function accesses to all necessary inputs during the map phase. In the reduce phase, all the nodes compute their assigned reduce functions using the gathered IVs.

Due to massive data exchange and limited communication bandwidth, the MapReduce-type system (MapReduce in short) often suffers from communication bottlenecks, i.e., the data shuffle phase consumes a significant portion of the overall task execution time. In order to reduce the communication overhead during the shuffling phase, Li et al. in [4] proposed a CDC scheme which achieves an optimal tradeoff between the computation load and communication load under a given output function assignment. The CDC scheme has also been widely studied in the other distributed computing scenarios. For example, CDC has been combined with maximum distance separable (MDS) codes to handle matrix–vector multiplication tasks and mitigate the impact of stragglers [5]. There are also many workers focusing on the stragglers in general functions [6,7], optimal resource allocation strategies [8,9], iterative computing and shuffling procedures [10,11], randomized file allocations [12], and cases with random network connectivity [13].

The CDC technique has been extended to wireless networks [14,15], where devices exchange information via wireless links. In such scenarios, owing to the decentralized nature of wireless networks, a central access point (AP) is typically required to facilitate data exchange, giving rise to uplink and downlink communication phases [16,17,18]. In real-world scenarios, we usually consider multiple APs. For example, a large place like a university campus needs to deploy multiple APs to realize campus network coverage. Moreover, a multi-AP system is able to distribute the load to multiple repeaters, thus reducing the risk of a single point of failure and improving fault tolerance.

This paper considers the CDC problem in a wireless (H,L)-combination network [19] which consists of *H* numbers of APs and K=HL computing workers. Each worker is connected to a distinct subset of *L* numbers of APs through wireless links. The APs act as intermediate hubs, facilitating the exchange of IVs among workers over wireless channels. Each worker is assigned a subset of the input files, processes them locally to compute IVs, and then transmits some IVs to its connected APs. The APs aggregate and broadcast the received information to other workers, ensuring that all workers obtain the necessary IVs to compute their respective output functions. This multi-AP structure provides enhanced reliability and robustness in the face of AP or node failures. We are interested in the tradeoff between the communication load and computation load (defined as the average number of nodes processing each input file), and aim to improve the overall efficiency of the distributed computing system by exploiting computation resources at workers and creating additional opportunities for parallelism and multicast transmission.

In this paper, we first obtain a baseline scheme by using CPDA. Then, by novelly characterizing the scheme from the view point of linear algebra, the problem of designing a CDC scheme for combination network is equivalent to constructing a special matrix. Finally, using CPDA, we obtain an improved CDC scheme which has a lower communication load than that of the baseline scheme, for which the computation load does not increase.

**Notations**: We denote the set of positive integers by $N^{+}$. For $n\in N^{+}$, the set {1,…,n} is denoted as [n], and |.| is used to represent the cardinality of a set or the length of a vector. Denote [H]L={A|A∈[H],|A|=L}, i.e., [H]L is the collection of subsets of [H] of size *L*.

## 2. System Model

**Figure 1:**
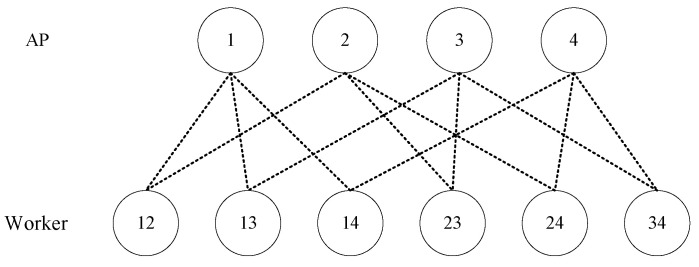
A combination network with H=4, L=2.

We consider a (H,L,M,N) distributed coded computing system for a combination network, where there are *N* input files $W=\{w_{n}\;|\;n\in \left[N\right]\}$, each of which has *B* bits, *H* APs H={1,2,…,H} without storage, *K* workers, each of which can store at most *M* files, and *K* output functions $Q=\{\phi_{q}\;|\;q\in \left[K\right]\}$ from *N* files, each of which is arranged to one worker. Each worker is connected to a unique *L*-subset of APs over the wireless channel. So K=HL and the user set can be written as K=[H]L. Each worker is connected to the other workers via these wireless channels through common APs. For instance, Figure 1 shows a combination network with H=4 and L=2. For each q∈[K], the output function $\phi_{q}$ maps the *N* input files into a streams of *U* bits, i.e., we have $\phi_{q}:F_{2^{B}}^{N}\to F_{2^{U}}$. The map function is defined as $g_{q}:F_{2^{B}}\to F_{2^{T}}$. For each n∈[N], the map function maps the input file $w_{n}$ into intermediate value (IV) $v_{q,n}=g_{q}\left(w_{n}\right)\in F_{2^{T}}$ of *T* bits. Similarly, for every q∈[K], we define the reduce function as $h_{q}:F_{2^{T}}^{N}\to F_{2^{U}}$, which maps the *N* IVs into a stream of *U* bits. Consequently, the output function $\phi_{q}\left(W\right)$ is given by

$$\phi_{q}\left(W\right)=h_{q}\left(v_{q,1},\ldots ,v_{q,N}\right)=h_{q}\left(g_{q}\left(w_{1}\right),\ldots ,g_{q}\left(w_{N}\right)\right)$$

as described in [4,18,20]. Thus, a (H,L,M,N) distributed coded computing scheme for a combination network consists of the following three phases:

•**Map Phase:** Each worker k∈K stores *M* files, denoted by $Z_{k}$. We assume that each file is stored the same number of times. Based on its local stored files, worker k∈K can compute all the IVs $\left\{v_{q,n}\;|\;w_{n}\in Z_{k},q\in \left[K\right]\right\}$.

•**Shuffle Phase:** Each worker k∈K is assigned to compute an output function $\phi_{q}$, where q∈[K]. In order to obtain all the IVs $\left\{v_{q,n}\;|\;n\in \left[N\right]\right\}$, the worker needs to request its required IVs from the other workers through their common APs. So, the communication process consists of two phases: uplink and downlink steps. Before the transmission begins, each worker encodes the IVs $v_{q,n}\in F_{2^{T}}$ as ${\tilde{v}}_{q,n}\in C$ using a randomized Gaussian encoding scheme. We assume that the transmission strategy is one-shot linear, i.e., each transmitted coded IV in a transmission could be decoded by any required worker and its local IVs. At each time slot *s*, in the uplink step, we denote the signal sent from the worker k∈K by $X_{k}\left(s\right)$ which satisfies the power constraint $E[|X_{i}\left(s\right){|}^{2}]\leq P$. Here, the worker can be silent, i.e., not send any data. Then, each AP h∈H receives the following signal:

(1) $$\begin{array}{c} y_{h}\left(s\right)=\sum_{k\in K}c_{k,h}X_{k}\left(s\right)+z_{h}\left(s\right), \end{array}$$

where $c_{k,h}$ and $z_{h}\left(s\right)$ denote the channel coefficients from worker *k* to AP *h* and the additive white Gaussian noise, respectively. We assume that $c_{k,h}$ is chosen from C if worker *k* is connected to AP *h*; otherwise, $c_{k,h}=0$. Additionally, $z_{h}\left(s\right)$ follows a normal distribution N(0,1) if worker *k* is connected to AP *h*; otherwise, $z_{h}\left(s\right)=0$.

In the downlink step, each AP h∈H sends the received coded signal $y_{h}\left(s\right)$ from its connected workers. The goal of the communication is to obtain all the IVs $v_{q,k}$ required by each worker k∈K, who is arranged to compute the output function $\phi_{q}$.

Specifically, each worker k∈K receives the following signal from its connected APs

(2) $$\begin{array}{c} Y_{k}\left(s\right)=\sum_{h\in H}c_{k,h}y_{h}\left(s\right)+z_{k}^{\prime }\left(s\right), \end{array}$$

where $z_{k}^{\prime }\left(s\right)$ is also the additive white Gaussian noise, and if *h* is connected to *k*, then $z_{k}^{\prime }\left(s\right)$ follows a normal distribution with N(0,1); otherwise, $z_{k}^{\prime }\left(s\right)=0$. We can omit the additive noise terms $z_{h}\left(s\right)$ and $z_{k}^{\prime }\left(s\right)$. This assumption is valid under the condition that the transmit power is sufficiently large to ensure a high signal-to-noise ratio (SNR).

•**Reduce Phase:** After receiving all the signals sent down by the AP, each worker k∈K can decode all its required IVs and compute the arranged output function $\phi_{q}\left(W\right)$.

**Definition 1.**  *The following two types of loads are used as criteria for evaluating the merits of a (H,L,M,N) distributed coded computing scheme for combination network:*

- ***Computation load*** *is defined as the total number of mapped files across the K workers, normalized by N, i.e., $r≜\frac{\sum_{k=1}^{K}\left|Z_{k}\right|}{N}$;*
- ***Communication load*** *consists of the uplink load $L_{u}≜\frac{\sum_{h=1}^{H}l_{h}^{u}}{KNT}$, where $l_{h}^{u}$ is the length of the message received by the AP h in the uplink phase, i.e., the total normalized number of bits received by the H APs in the uplink process, and the downlink load $L_{d}≜\frac{\sum_{h=1}^{H}l_{h}^{d}}{KNT}$ where $l_{h}^{d}$ is the length of the message sent by AP h in the downlink phase, i.e., the total normalized number of bits sent by the H APs in the downlink process.*

The authors in [21] introduced the CPDA to realize a coded caching scheme for a combination network. In this paper, we will use it to construct our schemes.

**Definition 2**  (CPDA)**.** *For any positive integers H*,* N*,* Z and L with L≤H and Z≤N*,* let K=HL*,* an N×K array P=(pn,L)n∈[N],L∈[H]L over [S]∪{∗} is called a (K,N,Z,S) CPDA if it satisfies the following conditions:*

*C1.*  *The symbol "∗" appears Z times in each column;*
*C2.*  *Each s∈[S] occurs at least once in the array;*
*C3.*  *For any two distinct entries $p_{n_{1},L_{1}}$ and $p_{n_{2},L_{2}}$ satisfying $p_{n_{1},L_{1}}=p_{n_{2},L_{2}}=s\in \left[S\right]$, then $p_{n_{1},L_{2}}=p_{n_{2},L_{1}}=*$;*
*C4.*  *For any s∈[S], the labels of all columns containing symbol s have a nonempty intersection.*

*If each integer occurs exactly g times in P*,* we call it g-regular CPDA.*

Let us take the following example to further illustrate the concept of CPDA.

**Example 1.**  *When H=N= 4 and Z=L= 2, let us consider the following array.*

$$\begin{array}{c} P=(\begin{array}{cccccc} 12 & 13 & 14 & 23 & 24 & 34\\ 1 & 3 & 4 & * & * & *\\ 2 & * & * & 3 & 4 & *\\ {}* & 2 & * & 1 & * & 4\\ {}* & * & 2 & * & 1 & 3 \end{array}). \end{array}$$

*Clearly each column has exactly Z= 2 stars, and each integer occurs at least once in P. So the conditions C1–2 hold. Let us consider the conditions C3–4. When s= 1, we can obtain the following subarray:*

$$\begin{array}{c} P^{\left(1\right)}=(\begin{array}{ccc} 12 & 23 & 24\\ 1 & * & *\\ {}* & 1 & *\\ {}* & * & 1 \end{array}). \end{array}$$

*Clearly, the entries*

$$\begin{array}{cc} \; & p_{1,\{2,3\}}=p_{1,\{2,4\}}=p_{3,\{1,2\}}=p_{3,\{2,4\}}=p_{4,\{1,2\}}=p_{4,\{2,3\}}=*,\\ \; & p_{1,\{1,2\}}=p_{3,\{2,3\}}=p_{4,\{2,4\}}=1. \end{array}$$

*Therefore*,* the above two symbols 1 satisfy condition C3. The column labels corresponding to each integer 1 are {1,2}*,* {2,3}*,* and {2,4}. Clearly*,* {1,2}∩{2,3}∩{2,4}={2}≠∅*,* which satisfy condition C4. Similarly, we can obtain the subarrays which only contain integers 2*,* 3*,* and 4*,* respectively and show that the conditions C3–4 hold. So*,* P is a (6,4,2,4) CPDA.*

There are many constructions of CPDAs in [21,22,23,24]. Here, we list some results which we will use in this paper as follows:

**Lemma 1**  ([21])**.** *For any positive integers H, L, and t satisfying L|H and t<K′=H−1L−1*,* there exists a (HL,LK′t*,* LK′−1t−1*,* HK′t+1) CPDA.*

**Lemma 2**  ([22])**.** *For any positive integers H,L,b,λ satisfying 0<L*,* b<H*,* λ<L*,* λ≤b and L+b−2λ<H*,* there exists a (HL,Hb,Hb−LλH−Lb−λ,HL+b−2λ·min{H−L−b+2λλ,L+b−2λL−λ}) CPDA.*

## 3. Main Results

In this section, the baseline scheme is introduced. Then, we characterize the CDC scheme from the view point of a coefficient matrix, thereby obtaining an optimized CDC scheme for the combination network. Finally, we use CPDA to derive the required coefficient matrix, which significantly simplifies the system design and enhances its performance. In addition, we demonstrate that the proposed scheme achieves a lower transmission load than the baseline scheme.

When each output function is computed by exactly one worker, the authors in [25] showed that the CDC problem is equivalent to the coded caching problem for device-to-device (D2D) networks. In addition, the authors in [26] proposed that a coded caching scheme for a shared link can be used to generate a coded caching scheme for a D2D network. Using the same method in [26], we can generate a CDC scheme for a combination network based on the CPDA and obtain the following result.

**Theorem 1**  (Baseline scheme)**.** *Given a g-(K,N,Z,S) CPDA P with g>1 and K=HL*,* there exists a (H,L,M,N) baseline coded distributed computing scheme for a (H,L)-combination network*,* with a computation load of $r=\frac{KZ}{N}$ and uplink and downlink communication loads of*

(3) $$\begin{array}{c} L_{u}=L_{d}=\frac{Sg}{KN(g-1)}. \end{array}$$

**Proof.**  We use the star positions in P to assign the files to each worker as follows. When $p_{n,k}=*$, worker *k* stores file $w_{n}$. So worker *k* stores the following files:

(4) $$Z_{k}=\left\{w_{n}|n\in \left[N\right],p_{n,k}=*\right\}.$$

In the map phase, the set of IVs computed locally by worker *k* is

(5) $$V_{k}=\left\{v_{q,n}|q\in \left[K\right],n\in \left[N\right],p_{n,k}=*\right\}.$$

According to Definition 1, the computation load is $r=\frac{\sum_{k=1}^{K}\left|Z_{k}\right|}{N}=\frac{KZ}{N}$.

Recall that each worker k∈K is assigned to compute the output function $\phi_{q_{k}}$. From (5), worker *k* contains the required IVs in $\left\{v_{q_{k},n}|p_{n,k}=*,n\in \left[N\right]\right\}$ and needs the other required IVs in $\left\{v_{q_{k},n}|p_{n,k}\neq *,n\in \left[N\right]\right\}$, each of which should be transmitted by the other workers.

Now, we will use the integers in P to design the transmission strategy in the shuffle phase. For each integer s∈[S], we assume that *s* occurs exactly *g* times in P. That is,

$$p_{n_{1},k_{1}}=p_{n_{2},k_{2}}=\ldots =p_{n_{g},k_{g}}=s.$$

Denote the set of workers associated with *s* as $U_{s}=\left\{k_{1},k_{2},\ldots ,k_{g}\right\}$ and their respective connected AP sets as

$$L=\{L_{k_{1}},L_{k_{2}},\ldots ,L_{k_{g}}\}.$$

By the condition C4 of Definition 2, there exists at least one integer, say $h_{s}$, satisfying

$$h_{s}\in L_{k_{1}}\cap L_{k_{2}}\cap \ldots \cap L_{k_{g}}.$$

Recall that in the map phase, each worker can encode the IV $v_{q,n}\in V_{k}$ into ${\tilde{v}}_{q,n}$ using a random Gaussian coding scheme, where ${\tilde{v}}_{q,n}$ has a size of *t* bits. To facilitate efficient transmission, each IV ${\tilde{v}}_{q,n}$ in $\left\{{\tilde{v}}_{q_{k_{1}},n_{1}},{\tilde{v}}_{q_{k_{2}},n_{2}},\ldots ,{\tilde{v}}_{q_{k_{g}},n_{g}}\right\}$ is divided into g−1 equal-sized packets. That is,

(6) $${\tilde{v}}_{q_{k},n}={\left({\tilde{v}}_{q_{k},n}^{\left(k_{i}\right)}\right)}_{k_{i}\in U_{s},k_{i}\neq k},$$

where ${\tilde{v}}_{q_{k},n}^{\left(k_{i}\right)}$ denotes the packet of ${\tilde{v}}_{q_{k},n}$ intended for worker $k_{i}$.

In the shuffle phase, each worker $k\in U_{s}$ transmits the following encoded information of length $\frac{t}{g-1}$ bits to the AP $h_{s}$

(7) $$X_{k}\left(s_{k}\right)=\sum_{p_{n_{i},k_{i}}=s,k_{i}\neq k}{\tilde{v}}_{q_{k_{i}},n_{i}}^{\left(k\right)},$$

where $s_{k}$ represents the sub-slot within time slot *s* allocated to worker $k\in U_{s}$ for transmitting its encoded data. Upon receiving the message $y_{h_{s}}\left(s_{k}\right)=c_{k,h_{s}}X_{k}\left(s_{k}\right)$ where the coefficient $c_{k,h_{s}}$ is the channel coefficient between worker *k* and AP $h_{s}$ and is chosen from C under the independent and identically distributed condition, the AP $h_{s}$ broadcasts it to all workers in $U_{s}\setminus \left\{k\right\}$. Consequently, each worker $k_{i}\in U_{s}\setminus \left\{k\right\}$ receives the message

(8) $$Y_{k_{i}}\left(s_{k}\right)=c_{k_{i},h_{s}}y_{h_{s}}\left(s_{k}\right)=c_{k_{i},h_{s}}c_{k,h_{s}}X_{k}\left(s_{k}\right).$$

In the reduce phase, each worker k∈K needs to compute the output function $\phi_{q_{k}}$. By Condition C3 of Definition 2 and Equation (5), each worker $k_{i}\in U_{s}\setminus \left\{k\right\}$ already possesses all IVs in $X_{k}\left(s_{k}\right)$ except ${\tilde{v}}_{q_{k_{i}},n_{i}}^{\left(k\right)}$. Therefore, each worker $k_{i}\in U_{s}$ can decode its desired IV ${\tilde{v}}_{q_{k_{i}},n_{i}}^{\left(k\right)}$ from $Y_{k_{i}}\left(s_{k}\right)$. After time slot *s*, each worker $k_{i}\in U_{s}$ obtains the IV ${\tilde{v}}_{q_{k_{i}},n_{i}}$. After all *S* time slots, each worker k∈K obtains all the missing IVs $\left\{v_{q_{k},n}|p_{n,k}\neq *,n\in \left[N\right]\right\}$, which are required to compute the output function $\phi_{q_{k}}$. With all the necessary IVs, each worker k∈K proceeds to compute $\phi_{q_{k}}$ and completes the reduce phase.

Now, let us consider the communication load. By Definition 1, the uplink and downlink loads are

(9) $$\begin{array}{cc} L_{u}=L_{d} & =\frac{1}{KNt}\sum_{s=1}^{S}\frac{gt}{\left(g-1\right)}=\frac{S}{KN}\frac{g}{\left(g-1\right)}. \end{array}$$

Then, the proof is completed. □

### 3.1. Main Idea

In this subsection, we characterize the CDC scheme under the combination network from a matrix perspective. Our task is to compute Q=K output functions, where each worker k∈K owns a portion of the input file denoted as $Z_{k}$.

**Map Phase:** For each mapped file $w_{n}\in Z_{k}$, worker *k* computes the IV $v_{q,n}$ for all *K* output functions. Consequently, the set of IVs available at worker k∈K is given by

(10) $$V_{k}=\left\{v_{q,n}|q\in \left[K\right],w_{n}\in Z_{k}\right\}.$$

The computation load in the map phase is given by $r=\frac{\sum_{k=1}^{K}\left|Z_{k}\right|}{N}.$

**Shuffle Phase:** The shuffle phase consists of two processes: (1) workers upload IVs to the AP via wireless channels, and (2) the AP broadcasts the received IVs to the connected workers. Before transmitting the information, each worker encodes the IV $v_{q,n}$ into ${\tilde{v}}_{q,n}$ using a random Gaussian coding scheme. During time slot *s*, a set of workers $U_{s}=\left\{k_{1},k_{2},\ldots ,k_{g}\right\}$ uploads IVs to AP $h_{s}$. Assuming that the signal sent by worker $k_{i}\in U_{s}$ at time slot *s* is denoted as $X_{k_{i}}\left(s\right)$, the signal received by AP $h_{s}$ can be expressed as

(11) $$y_{h_{s}}\left(s\right)=C_{s}X_{s}=\left[\begin{array}{cccc} c_{k_{1},h_{s}} & c_{k_{2},h_{s}} & \ldots & c_{k_{g},h_{s}} \end{array}\right]\left[\begin{array}{c} X_{k_{1}}\left(s\right)\\ X_{k_{2}}\left(s\right)\\ \vdots \\ X_{k_{g}}\left(s\right) \end{array}\right],$$

where $C_{s}$ is the channel coefficient matrix for time slot *s*, and $X_{s}$ is the transmit signal vector for time slot *s*. To design $X_{k_{i}}\left(s\right)$, we define $\alpha_{k_{i}}=\left(a_{k_{i},n_{1}},a_{k_{i},n_{2}},\ldots ,a_{k_{i},n_{g}}\right)$ as the encoding coefficients for worker $k_{i}$. Then,

(12) $$X_{s}=\left[\begin{array}{c} X_{k_{1}}\left(s\right)\\ X_{k_{2}}\left(s\right)\\ \vdots \\ X_{k_{g}}\left(s\right) \end{array}\right]=\left[\begin{array}{c} \alpha_{k_{1}}\\ \alpha_{k_{2}}\\ \vdots \\ \alpha_{k_{g}} \end{array}\right]\left[\begin{array}{c} {\tilde{v}}_{q_{k_{1}},n_{1}}\\ {\tilde{v}}_{q_{k_{2}},n_{2}}\\ \vdots \\ {\tilde{v}}_{q_{k_{g}},n_{g}} \end{array}\right]=\left[\begin{array}{ccccc} a_{k_{1},n_{1}} & a_{k_{1},n_{2}} & \ldots & a_{k_{1},n_{g}}\\ a_{k_{2},n_{1}} & a_{k_{2},n_{2}} & \ldots & a_{k_{2},n_{g}}\\ \vdots & \vdots & \; & \vdots & \\ a_{k_{g},n_{1}} & a_{k_{g},n_{2}} & \ldots & a_{k_{g},n_{g}} \end{array}\right]\left[\begin{array}{c} {\tilde{v}}_{q_{k_{1}},n_{1}}\\ {\tilde{v}}_{q_{k_{2}},n_{2}}\\ \vdots \\ {\tilde{v}}_{q_{k_{g}},n_{g}} \end{array}\right]=A_{s}{\tilde{V}}_{s},$$

where $A_{s}$ is the coefficient matrix for time slot *s*, and ${\tilde{V}}_{s}$ is the vector of encoded IVs. Thus, the message received by AP $h_{s}$ is

(13) $$y_{h_{s}}\left(s\right)=C_{s}A_{s}{\tilde{V}}_{s}.$$

By designing the coefficient matrix $A_{s}$, we can control the IVs uploaded by workers to AP $h_{s}$, completing the uplink process.

Next, we describe the downlink process. In time slot *s*, AP $h_{s}$ broadcasts the received message $y_{h_{s}}\left(s\right)$ to its connected workers. Each worker $k_{i}\in U_{s}$ then receives

(14) $$\begin{array}{c} Y_{k_{i}}\left(s\right)=c_{k_{i},h_{s}}y_{h_{s}}\left(s\right). \end{array}$$

Using the IVs it already possesses, worker $k_{i}$ can decode the required information from $Y_{k_{i}}\left(s\right)$.

After *S* time slots, all *K* workers acquire the missing IVs required for the reduce function. Specifically, each worker k∈K obtains

$$\{v_{q,n}|q=q_{k},n\in \left[N\right],w_{n}\notin Z_{k}\}.$$

By combining their own IVs $V_{k}$, each worker now possesses all the necessary information to compute the output function.

Since the messages in both the uplink and downlink processes are $y_{h_{s}}\left(s\right)$, and assuming each IV has a size of *t* bits, the communication load per time slot is *t* bits. According to Definition 1, the total uplink and downlink communication loads are $L_{u}=L_{d}=\frac{St}{KNt}=\frac{S}{KN}.$

**Reduce Phase:** After the shuffle phase, each worker k∈K computes the output function $\phi_{q_{k}}$ using all the IVs they possess.

**Theorem 2.**  *Given an (K,N,Z,S) CPDA with K=HL*,* there exists a (H,L,M,N) coded distributed computing scheme under a combination network*,* with a computation load of $r=\frac{KZ}{N}$ and its uplink and downlink communication loads given by*

(15) $$\begin{array}{c} L_{u}=L_{d}=\frac{S}{KN}. \end{array}$$

**Proof.**  We use a (K,N,Z,S) CPDA to design both the input file assignment and the coefficient matrix for the shuffle phase.

Before the computation task begins, the input file assigned to each worker k∈K is

(16) $$Z_{k}=\left\{w_{n}|n\in \left[N\right],p_{n,k}=*\right\}.$$

In the map phase, each worker maps the input file it owns into *K* IVs $v_{q,n}$. Thus, the IVs at worker k∈K are

(17) $$V_{k}=\left\{v_{q,n}|q\in \left[K\right],n\in \left[N\right],p_{n,k}=*\right\}.$$

Since each worker is allocated *Z* input files, the computation load according to Definition 1 is $r=\frac{\sum_{k=1}^{K}\left|Z_{k}\right|}{N}=\frac{KZ}{N}.$

Before the shuffle phase, each worker encodes the IV $v_{q,n}$ into ${\tilde{v}}_{q,n}$ using a random Gaussian coding scheme, where ${\tilde{v}}_{q,n}$ has a size of *t* bits. In the shuffle phase, we consider an integer s∈[S] in the CPDA P, and suppose *s* occurs *g* times in P. Let $p_{n_{1},k_{1}}=p_{n_{2},k_{2}}=\ldots =p_{n_{g},k_{g}}=s$. The set of workers involved in time slot *s* is $U_{s}=\left\{k_{1},k_{2},\ldots ,k_{g}\right\}$, and the APs connected to all workers in $U_{s}$ are denoted as $I_{s}=\left\{h|h\in H,h\;\mathrm{is}\;\mathrm{connected}\;\mathrm{to}\;\mathrm{all}\;k_{i}\in U_{s}\right\}$. An arbitrary AP $h_{s}\in I_{s}$ is selected to handle the uplink and downlink communication in time slot *s*.

In time slot *s*, we design the message received by AP $h_{s}$ as

(18) $$y_{h_{s}}\left(s\right)=\sum_{i=1}^{g}{\tilde{v}}_{q_{k_{i}},n_{i}}=\left[\begin{array}{cccc} 1 & 1 & \ldots & 1 \end{array}\right]\left[\begin{array}{c} {\tilde{v}}_{q_{k_{1}},n_{1}}\\ {\tilde{v}}_{q_{k_{2}},n_{2}}\\ \vdots \\ {\tilde{v}}_{q_{k_{g}},n_{g}} \end{array}\right].$$

Let

$${\tilde{V}}_{s}=\left[\begin{array}{c} {\tilde{v}}_{q_{k_{1}},n_{1}}\\ {\tilde{v}}_{q_{k_{2}},n_{2}}\\ \vdots \\ {\tilde{v}}_{q_{k_{g}},n_{g}} \end{array}\right].$$

From (13) and (18), we need to design the coefficient matrix $A_{s}$ such that

(19) $$\left[\begin{array}{cccc} c_{k_{1},h_{s}} & c_{k_{2},h_{s}} & \ldots & c_{k_{g},h_{s}} \end{array}\right]A_{s}=\left[\begin{array}{cccc} 1 & 1 & \ldots & 1 \end{array}\right].$$

Since $p_{k_{i},n_{i}}\neq *$, by (17), worker $k_{i}$ does not possess the IV ${\tilde{v}}_{q_{k_{i}},n_{i}}$. Thus, $a_{k_{i},n_{i}}=0$, and (19) can be written as

(20) $$\left[\begin{array}{cccc} c_{k_{1},h_{s}} & c_{k_{2},h_{s}} & \ldots & c_{k_{g},h_{s}} \end{array}\right]\left[\begin{array}{cccc} 0 & a_{k_{1},n_{2}} & \ldots & a_{k_{1},n_{g}}\\ a_{k_{2},n_{1}} & 0 & \ldots & a_{k_{2},n_{g}}\\ \vdots & \vdots & \; & \vdots \\ a_{k_{g},n_{1}} & a_{k_{g},n_{2}} & \ldots & 0 \end{array}\right]=\left[\begin{array}{cccc} 1 & 1 & \ldots & 1 \end{array}\right].$$

By linear algebra, i.e., given the row vector $\left(c_{k_{1},h_{s}},c_{k_{2},h_{s}},\ldots ,c_{k_{g},h_{s}}\right)$, we can always obtain a non-zero column vector $a_{i}$ of $A_{s}$ such that the *i*th coordinate is zero. So, we can always obtain the coefficient matrix $A_{s}$ in (20).

In the uplink phase, each worker $k_{i}\in U_{s}$ transmits the encoded information $X_{k_{i}}\left(s\right)=\alpha_{k_{i}}{\tilde{V}}_{s}$ to AP $h_{s}$. In the downlink phase, AP $h_{s}$ broadcasts $y_{h_{s}}\left(s\right)$ to all workers in $U_{s}$. From Condition C3 of Definition 2 and Equation (17), each worker $k_{i}\in U_{s}$ already possesses all IVs in $y_{h_{s}}\left(s\right)$ except ${\tilde{v}}_{q_{k_{i}},n_{i}}$. Thus, each worker $k_{i}\in U_{s}$ can decode its desired IV ${\tilde{v}}_{q_{k_{i}},n_{i}}$ from $y_{h_{s}}\left(s\right)$. After *S* time slots, all *K* workers obtain the missing IVs necessary for computing the reduce function.

Since the size of each IV ${\tilde{v}}_{q,n}$ is *t* bits, the communication load per time slot is *t* bits. The total uplink and downlink communication loads are

(21) $$L_{u}=L_{d}=\frac{St}{KNt}=\frac{S}{KN}.$$

In the reduce phase, each worker k∈K computes the output function $\phi_{q_{k}}(w_{1},w_{2},\ldots ,w_{N})$. Since $\phi_{q_{k}}\left(w_{1},w_{2},\ldots ,w_{N}\right)=h_{k}\left(v_{q_{k},1},v_{q_{k},2},\ldots ,v_{q_{k},N}\right)$, worker *k* utilizes the IVs of all *N* files for the $q_{k}$-th output function. After the shuffle phase, each worker k∈K is given the IVs needed to compute $\phi_{q_{k}}$. Therefore, all workers can compute their assigned output functions. □

### 3.2. Performance Evaluation

In this subsection, let us consider the performance of the proposed schemes by applying the CPDA in Lemmas 1 and 2 to Theorems 1 and 2, respectively. The resulting schemes are summarized in Table 1 and Table 2 respectively. In the following, let us consider the computation load and communication load of these schemes.

**Figure 2:**
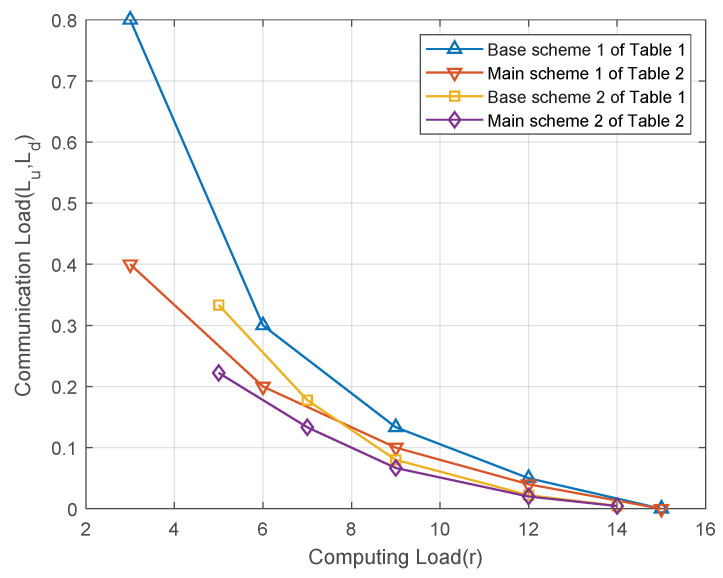
The relationship between the computation load and the communication load for the schemes in Table 1 and Table 2 is illustrated for a distributed computing system with K=15 computing nodes and Q=K=15 output functions under the (6,2)-combination network.

Figure 2 illustrates the relationship between the computation load and the communication load for the schemes in Table 1 and Table 2. Let us consider the distributed computing system with K=15 computing nodes and Q=K=15 output functions under the (6,2)-combination network. As shown in Figure 2, when the same class of CPDA is used, the main scheme of Theorem 2 achieves a lower communication load than the base scheme of Theorem 1 for the same computation load. This improvement results from the main scheme’s more efficient utilization of the multicast gain, which reduces the communication load.

### 3.3. Example for Achievable Scheme

We consider a combinatorial network with H=3, L=2, where K=3 and N=3. The tasks to be computed by the 3 workers are $\phi_{1}$, $\phi_{2}$, and $\phi_{3}$, respectively. The workers are denoted as K={12,13,23}, and the input files are denoted as $W=\{w_{1},w_{2},w_{3}\}$. Before starting the task, the input files are assigned as follows: $Z_{12}=\left\{w_{1}\right\}$ to worker 12, $Z_{13}=\left\{w_{2}\right\}$ to worker 13, and $Z_{23}=\left\{w_{3}\right\}$ to worker 23.

#### 3.3.1. Map Phase

In the map phase, each worker maps each assigned file into 3 IVs. Specifically:

- Worker 12 owns $V_{12}=\left\{v_{1,1},v_{2,1},v_{3,1}\right\}$ but lacks $\left\{v_{1,2},v_{1,3}\right\}$.
- Worker 13 owns $V_{13}=\left\{v_{1,2},v_{2,2},v_{3,2}\right\}$ but lacks $\left\{v_{2,1},v_{2,3}\right\}$.
- Worker 23 owns $V_{23}=\left\{v_{1,3},v_{2,3},v_{3,3}\right\}$ but lacks $\left\{v_{3,1},v_{3,2}\right\}$.

#### 3.3.2. Shuffle Phase

After the map phase, the shuffle phase begins, where workers exchange IVs via the APs. Each worker encodes the IVs $v_{q,n}$ into ${\tilde{v}}_{q,n}$ using a randomized Gaussian coding scheme to ensure reliable transmission over the wireless channel.

- **Time Slot 1:** Workers 12 and 13 send $X_{12}\left(1\right)=\frac{1}{c_{12,1}}{\tilde{v}}_{2,1}$ and $X_{13}\left(1\right)=\frac{1}{c_{13,1}}{\tilde{v}}_{1,2}$ to AP 1 simultaneously. AP 1 receives the message $y_{1}\left(1\right)={\tilde{v}}_{1,2}+{\tilde{v}}_{2,1}$, completing the uplink process. AP 1 then broadcasts $y_{1}\left(1\right)$ to workers 12 and 13, who receive $Y_{12}\left(1\right)=c_{12,1}\left({\tilde{v}}_{1,2}+{\tilde{v}}_{2,1}\right)$ and $Y_{13}\left(1\right)=c_{13,1}\left({\tilde{v}}_{1,2}+{\tilde{v}}_{2,1}\right)$, respectively.
- **Time Slot 2:** Workers 12 and 23 send $X_{12}\left(2\right)=\frac{1}{c_{12,2}}{\tilde{v}}_{3,1}$ and $X_{23}\left(2\right)=\frac{1}{c_{23,2}}{\tilde{v}}_{1,3}$ to AP 2 simultaneously. After the uplink and downlink phases, workers 12 and 23 receive $Y_{12}\left(2\right)=c_{12,2}\left({\tilde{v}}_{1,3}+{\tilde{v}}_{3,1}\right)$ and $Y_{23}\left(2\right)=c_{23,2}\left({\tilde{v}}_{1,3}+{\tilde{v}}_{3,1}\right)$, respectively.
- **Time Slot 3:** Workers 13 and 23 send $X_{13}\left(3\right)=\frac{1}{c_{13,3}}{\tilde{v}}_{3,2}$ and $X_{23}\left(3\right)=\frac{1}{c_{23,3}}{\tilde{v}}_{2,3}$ to AP 3 simultaneously. After the uplink and downlink phases, workers 13 and 23 receive $Y_{13}\left(3\right)=c_{13,3}\left({\tilde{v}}_{2,3}+{\tilde{v}}_{3,2}\right)$ and $Y_{23}\left(3\right)=c_{23,3}\left({\tilde{v}}_{2,3}+{\tilde{v}}_{3,2}\right)$, respectively.

#### 3.3.3. Reduce Phase

In the reduce phase, each worker decodes the missing IVs:

- Worker 12 solves ${\tilde{v}}_{1,2}$ and ${\tilde{v}}_{1,3}$ from $Y_{12}\left(1\right)$ and $Y_{12}\left(2\right)$ using its own ${\tilde{v}}_{2,1}$ and ${\tilde{v}}_{3,1}$.
- Worker 13 solves ${\tilde{v}}_{2,1}$ and ${\tilde{v}}_{2,3}$ from $Y_{13}\left(1\right)$ and $Y_{13}\left(3\right)$ using its own ${\tilde{v}}_{1,2}$ and ${\tilde{v}}_{3,2}$.
- Worker 23 solves ${\tilde{v}}_{3,1}$ and ${\tilde{v}}_{3,2}$ from $Y_{23}\left(2\right)$ and $Y_{23}\left(3\right)$ using its own ${\tilde{v}}_{1,3}$ and ${\tilde{v}}_{2,3}$.

After obtaining all the required IVs, each worker computes its assigned output function.

In this example, the computation load is $r=\frac{1+1+1}{3}=1$. Assuming each IV ${\tilde{v}}_{q,n}$ has a size of *t* bits, the uplink and downlink loads are calculated as $L_{u}=L_{d}=\frac{3t}{3\times 3t}=\frac{1}{3}$.

## 4. Conclusions

In this paper, we studied the coded distributed computing for (H,L)-combination networks and provided a comprehensive framework for constructing CDC scheme from the view point of linear algebra. Then, by using CPDAs, we proposed two classes of CDC schemes for the combination network. Finally by comparisons, we showed that the proposed schemes have lower communication loads than the baseline schemes.

## Figures and Tables

**Table 1:** The baseline schemes in Theorem 1.

Scheme	*r*	$L_{u},L_{d}$	Original
base scheme 1	$\frac{tH}{L}$	$\frac{K^{\prime }-t}{tK^{\prime }}$	Lemma 1
base scheme 2	HL−bλH−bL−λ	HL+b−2λ·H−L−b+2λλL+b−2λL−λHLHb(max(H−L−b+2λλ,L+b−2λL−λ)−1)	Lemma 2

**Table 2:** The main schemes in Theorem 2.

Scheme	*r*	$L_{u},L_{d}$	Original
main scheme 1	$\frac{tH}{L}$	$\frac{K^{\prime }-t}{\left(t+1\right)K^{\prime }}$	Lemma 1
main scheme 2	HL−bλH−bL−λ	HL+b−2λ·min(H−L−b+2λλ,L+b−2λL−λ)HLHb	Lemma 2

## Data Availability

The data are available upon request from the corresponding author.
